# Novel Identified HLA-A*0201-Restricted Hantaan Virus Glycoprotein Cytotoxic T-Cell Epitopes Could Effectively Induce Protective Responses in HLA-A2.1/K^b^ Transgenic Mice May Associate with the Severity of Hemorrhagic Fever with Renal Syndrome

**DOI:** 10.3389/fimmu.2017.01797

**Published:** 2017-12-12

**Authors:** Kang Tang, Linfeng Cheng, Chunmei Zhang, Yusi Zhang, Xuyang Zheng, Yun Zhang, Ran Zhuang, Boquan Jin, Fanglin Zhang, Ying Ma

**Affiliations:** ^1^Department of Immunology, The Fourth Military Medical University, Xi’an, China; ^2^Department of Microbiology, The Fourth Military Medical University, Xi’an, China; ^3^Department of Infectious Diseases, Tangdu Hospital, The Fourth Military Medical University, Xi’an, China

**Keywords:** Hantaan virus, hemorrhagic fever with renal syndrome, HLA-A*0201, cytotoxic T-cell epitope, cytotoxic T-cell response, peptide vaccine, HLA-A2.1/K^b^ transgenic mice

## Abstract

Hantaan virus (HTNV) infections can cause severe hemorrhagic fever with renal syndrome (HFRS) in humans, which is associated with high fatality rates. Cytotoxic T cell (CTL) responses contribute to virus elimination; however, to date, HLA class I allele-restricted HTNV glycoprotein (GP) epitopes recognized by CTLs have not been reported, limiting our understanding of CTL responses against HTNV infection in humans. In this study, 34 HTNV GP nine-mer epitopes that may bind to HLA-A*0201 molecules were predicted using the BIMAS and SYFPEITHI database. Seven of the epitopes were demonstrated to bind to HLA-A*0201 molecules with high affinity *via* the T2 cell binding assay and were successfully used to synthesize peptide/HLA-A*0201 tetramers. The results of tetramer staining showed that the frequencies of each epitope-specific CTL were higher in patients with milder HFRS, which indicated that the epitopes may induce protective CTL responses after HTNV infection. IFN-γ-enzyme-linked immunospot analysis further confirmed the immunoreactivity of epitopes by eliciting epitope-specific IFN-γ-producing CTL responses. In an HTNV challenge trial, significant inhibition of HTNV replication characterized by lower levels of antigens and RNA loads was observed in major target organs (liver, spleen, and kidneys) of HLA-A2.1/K^b^ transgenic mice pre-vaccinated with nonapeptides VV9 (aa8–aa16, VMASLVWPV), SL9 (aa996–aa1004, SLTECPTFL) and LL9 (aa358–aa366, LIWTGMIDL). Importantly, LL9 exhibited the best ability to induce protective CTL responses and showed a prominent effect on the kidneys, potentially preventing kidney injury after HTNV infection. Taken together, our results highlight that HTNV GP-derived HLA-A*0201-restricted epitopes could elicit protective CTL responses against the virus, and that epitope LL9 functions as an immunodominant protective epitope that may advance the design of safe and effective CTL-based HTNV peptide vaccines for humans.

## Introduction

Hantaviruses are enveloped negative-sense single-stranded RNA viruses that belong to the *Bunyaviridae* family, which are pathogenic to humans and are having a growing impact on global public health (1). In Europe and Asia, Dobrava-Belgrade virus (DOBV), Puumala virus (PUUV), Seoul virus (SEOV), and Hantaan virus (HTNV) infection can cause hemorrhagic fever with renal syndrome (HFRS) in humans. By contrast, in North and South American, Sin Nombre virus (SNV), Andes virus (ANDV), and New York virus are major pathogenic species that lead to hantavirus cardiopulmonary syndrome (HCPS) (also called hantavirus pulmonary syndrome, HPS) in humans (2–4). Each of the species gives rise to diseases with a broad spectrum of outcomes, ranging from asymptomatic infection to acute fever and hemorrhage, and even life-threatening shock and acute kidney injury (AKI) or acute respiratory injury (ARI) (5–7). In China, HTNV is the common pathogenic species causing severe HFRS. A total of 1,625,002 cases were reported during 1950–2014, accounting for approximately 90% of the total global cases, with a case-fatality rate as high as 15% (5, 8). In the United States, approximately 4,000 HCPS cases have been reported since 1993 and showed a higher case-fatality rate of approximately 36% (3, 9). Of note, rapid evolution and genetic reassortment between different hantavirus strains promote novel hantavirus expansion and the emergence of new epidemic characteristics, which introduces more challenges to prevent and control infection (10–14). Due to the limited understanding of the disease pathogenesis, no specific therapy is currently used. Vaccination is the most appropriate way to protect humans against virus infection, and although several vaccines have been generated by inactivation of the hantavirus, only a few of them are commercially produced and licensed for application in humans (8). In China, individuals immunized with HTNV-inactivated vaccine may still develop HFRS disease (15–17). There remains an urgent need to generate more effective vaccines to better prevent HTNV infection.

The HTNV genome consists of S, M, and L segments, which encode nucleocapsid protein (NP), precursor of Gn and Gc glycoproteins (GP) and RNA-dependent RNA polymerase (RdRp), respectively (13). The two structural proteins NP and GP give rise to the primary antigenicity of HTNV and are responsible for the vigorous humoral and cellular immune responses. Our previous results showed that T-cell responses during the acute phase of HFRS in patients are characterized by multifunctional T helper (Th) cell responses and vigorous cytotoxic T cell (CTL) responses (18–21). Patients with mild/moderate severity show vigorous HTNV-specific CD4^+^ and CD8^+^ T-cell responses against HTNV, while patients with severe/critical severity tend to have weak T-cell responses (19–21). Araki el al. found that HTNV infection was maintained in the presence of high titers of neutralizing antibodies, indicating that antibodies alone cannot eliminate the virus. Notably, NP was undetectable after adoptive transfer of HTNV-specific CD8^+^ T cells, suggesting that HTNV-specific CD8^+^ T cells are the major effector cells against the virus and contribute to the clearance of HTNV (22, 23). Moreover, individuals who had been infected with PUUV or HTNV maintained virus-specific memory CTLs many years after acute infection, revealing the importance of CTLs in preventing a second infection (24–26). Therefore, further identification of the CTL epitopes and exploration of the mechanism of CTL responses against HTNV infection are very desirable for the development of an effective vaccine for HFRS.

To date, most HTNV-specific CTL epitopes identified are distributed on NP (6, 27, 28). Early research identified three CTL epitopes on HTNV NP (25, 29). Recently, we also reported eight HTNV NP-specific CTL epitopes that were restricted by various HLA alleles including A2, A11, A24, B7, and B35 (6, 20, 30). It has been demonstrated that GP are the major antigens involved in the induction of neutralizing antibodies and play an important role in eliciting humoral immune responses to protect infected animals and humans against lethal hantavirus infection (17, 31). However, Kilpatrick et al. discovered that patients with HPS during early SNV infection showed higher frequencies of Gc epitope-specific CD8^+^ T cells than those of NP epitope-specific CD8^+^ T cells (32). Tobias et al. found that ANDV Gn-derived peptides were the immunodominant epitopes eliciting specific CD8^+^ T responses compared with those from NP and Gc. While NP-specific responses declined, Gn-specific responses remained readily detectable up to 13 years after infection (33). Hence, we assumed that HTNV GP-derived peptides may function as epitopes to induce effective CTL responses against HTNV infection. Recently, an HTNV Gn-derived H-2K^b^-restricted CTL epitope (ITSLFSLL) was identified (34), confirming our hypothesis that there are specific CTLs against HTNV GP-derived epitopes. However, H-2K^b^-restricted CTL epitopes may not induce parallel responses in humans, screaming HLA class I allele-restricted epitopes could provide additional help for better understanding of the recognition of HTNV by human CTLs. Importantly, Chinese Han populations in different regions have a similar allelic diversity, among which HLA-A*02 is the most common HLA class I allele (35, 36). Hence, the identification of HLA-A*02-restricted HTNV GP-specific CTL epitopes could be a promising, broadly applicable approach to the prevention of HTNV infection.

In the present study, we identified seven novel HTNV GP-derived HLA-A*0201-restricted nine-mer CTL epitopes and demonstrated that these epitopes could induce specific IFN-γ-producing CTL responses *in vitro*. Importantly, three epitopes, which were chosen to immunize HLA-A2.1/K^b^ transgenic (Tg) mice, could elicit immune responses to inhibit HTNV replication *in vivo*. These findings provide crucial information to characterize T-cell immunity against HTNV infection and may advance the design of safe and effective HTNV peptide vaccines, as well as the diagnosis and immuno-targeting of HFRS.

## Materials and Methods

### Ethics Statement

The study was approved by the Institutional Review Board of the Fourth Military Medical University, and all enrolled patients or their guardians signed an informed consent form before their blood was collected. The animal test was performed in strict accordance with the recommendation in the Guide for the Care and Use of Laboratory Animals of the National Health and Medical Research Council of China. The protocol was approved by the Committee on the Ethics of Animal Experiments of the Fourth Military Medical University under license number XJYYLL-2014437. All animal test procedures were performed under sodium pentobarbital anesthesia to minimize suffering.

### Patients

A total of 45 HLA-A*02^+^ HFRS patients infected with HTNV were enrolled in the study at the Department of Infectious Diseases at Tangdu Hospital of the Fourth Military Medical University (Xi’an, China). HTNV infection was confirmed *via* serological testing of immunoglobulin M and IgG in serum specimens. The clinical information for the enrolled patients is summarized in Table 1.

**Table 1:** Information for the patients with hemorrhagic fever with renal syndrome in this study.

	Mild/moderate	Severe/critical

Demographic characteristics
Patient number	22	23
Age (years)	40 (23–58)	46 (37–62)
Male/female	17/5	20/3

**Clinical parameters at acute phase**

Peak white blood cell count (× 10^9^/L)	8.35 (6.22–16.04)	13.79 (9.30–24.16)
Nadir platelet count (× 10^9^/L)	60 (47–93)	33 (13–46)
Peak blood urea nitrogen (mmol/L)	6.58 (5.32–16.51)	22.40 (14.38–31.45)
Peak serum creatinine (μmol/L)	115.00 (79.00–312.70)	343.20 (183.00–412.00)

*Values represent medians with the corresponding interquartile range*.

According to the diagnostic criteria from the Prevention and Treatment Strategy of HFRS described by the Ministry of Health, the People’s Republic of China, the severity degree of HFRS disease could be classified into four clinical types: mild, moderate, severe, and critical (21). (1) Mild: mild renal failure without an obvious oliguric stage; (2) moderate: obvious symptoms of effusion (bulbar conjunctiva), uremia, hemorrhage (skin and mucous membrane), and renal failure with a typical oliguric stage; (3) severe: severe effusion (bulbar conjunctiva and either pleura or peritoneum), uremia, hemorrhage (skin and mucous membrane), and renal failure with oliguria (urine output, 50–500 mL/day) for ≤5 days or anuria (urine output, <50 mL/day) for ≤2 days; (4) critical: ≥1 of the following symptoms during severe disease, visceral hemorrhage, refractory shock, heart failure, pulmonary edema, brain edema, severe secondary infection, and severe renal failure with oliguria (urine output, 50–500 mL/day) for >5 days, anuria (urine output, <50 mL/day) for >2 days, or a blood urea nitrogen level of >42.84 mmol/L. The patients who had other kidney diseases, diabetes, cardiovascular diseases, autoimmune diseases, hematological diseases, viral hepatitis, and other liver diseases were excluded from this study.

To ensure the sample size in some statistical analyses, we combined the patients according to the disease severity into mild/moderate and severe/critical groups for comparison. In this case, the number of patients with a severity degree of mild/moderate and severe/critical was 22 and 23, respectively. According to the clinical observation, the illness could be divided into the acute phase (the phase within 8 days from fever onset to the early oliguric stage) and the convalescent phase (diuretic and convalescent stages).

### Sample Collection

Peripheral blood samples were intravenously collected from 45 hospitalized HFRS patients and 18 HLA-A*02^+^ subjects who were seronegative for HTNV [normal controls (NC)]. Peripheral blood mononuclear cells (PBMCs) were isolated from the blood samples using standard Ficoll-Hypaque (Sigma-Aldrich, MO, USA) density gradient centrifugation. The PBMCs were then stained with fluorescein phycoerythrin (PE)-labeled anti-human HLA-A*02 monoclonal antibody (mAb) (Clone BB7.2; BioLegend) to test whether the HLA-A*02 was expressed by flow cytometry (FACScan; BD Biosciences). HLA-A*02^+^ PBMCs were selected and applied in the following assays.

### HTNV Viruses and HFRS Vaccine

The HTNV 76-118 strain was kindly provided by the Department of Microbiology of our university. The commercial HFRS inactivated vaccine (YOUERJIAN^®^, Zhejiang Tianyuan Bio-Pharmaceutical Co., Ltd., China) was derived from a mixture of both HTNV and SEOV and was provided as a bivalent and purified HFRS vaccine.

### Epitope Prediction and Peptide Synthesis

The Bioinformatics and Molecular Analysis Section (BIMAS) (^1^) HLA Peptide Binding Predictions was derived experimentally from the measurements of half-time dissociation rates of peptide–HLA complexes (37). The SYFPEITHI database^2^ contains a collection of MHC class I and class II ligands and peptide motifs of humans and other species (38, 39). Both BIMAS and SYFPEITHI were used to predict HLA-A*0201-restricted HTNV GP nine-mer epitopes by inputting the 1,135 amino acid sequence of the HTNV 76-118 strain (GenBank accession number: P08668.1) into the databases under the analysis options of HLA-A*0201 and nine-mer peptide. Based on the results predicted by BIMAS and SYFPEITHI, nine-mer peptides of HTNV GP were synthesized at 90% purity as assessed by high-performance liquid chromatography and mass spectrometry (CL Bio-scientific, Xi’an, China). Peptide VY9 (aa131–aa139, VPILLKALY) on HTNV NP confirmed to be the CTL epitope restricted by HLA-B*3501 in our previous study was synthesized as an uncorrelated peptide control (20). The CTL epitope FA9 (aa129–aa137, FVVPILLKA) on HTNV NP restricted by HLA-A*0201 was synthesized as a positive peptide control (6, 20). All the peptides were stored at a 1 mM concentration at −70°C and repeated freeze–thawing was avoided.

### T2 Cell Binding Assay

The lymphoblast cell line T2 (174 × CEM. T2) was purchased from the American Type Culture Collection (cat. no. CRL-1992™; Manassas, VA, USA). T2 cells are transporter-associated cells with antigen processing (TAP)-deficient and defective for endogenous MHC class I presentation. Although T2 cells express a very low level of the HLA-A*0201 molecule under normal culture conditions, they express HLA-A*0201 molecules at much higher levels after binding to appropriate peptides that stabilize the expression of HLA-A*0201 on the cell surface (40). To further determine the binding capability of predicted HTNV GP nonapeptides to HLA-A*0201 molecules, the T2 cell binding assay was performed as described elsewhere (41). Briefly, T2 cells were incubated with 50 µmol/L peptides and 1 µmol/L human β2-microglobulin (β2 m, Sigma) in serum-free RPMI 1640 medium for 18 h at 37°C with 5% CO_2_. The expression of HLA-A*0201 molecules on the surface of T2 cells was then determined by staining with PE-labeled anti-HLA-A*02 mAb (Clone BB7.2; BioLegend) and detected by flow cytometry (FACScan; BD Biosciences). The results are presented as the fluorescence index (FI), which was determined as follows: FI = (mean PE fluorescence with the given peptide − mean PE fluorescence without peptide)/(mean PE fluorescence without peptide) (6). FI ≥ 1 represents high-affinity peptides, indicating that the stable combination of these peptides with HLA-A*0201 molecules on the surface of T2 cells could increase the mean fluorescence of the HLA-A*0201 molecules by at least onefold.

### Peptide/HLA-A*0201 Tetramer Staining

PE-labeled HLA-A*0201 tetramers refolded separately with the HTNV GP nonapeptides were customized by QuantoBio (Beijing, China). The refolded peptides were screened as HLA-A*0201 high-affinity HTNV GP peptides in the T2 cell binding assay. The PBMCs of the patients were stained with each PE-labeled HLA-A*0201 tetramer for 10 min at room temperature, and subsequently stained with FITC-labeled anti-human CD3 mAb (Clone HIT3a; BioLegend) and PerCP-Cy5.5-labeled anti-human CD8 mAb (Clone HIT8a; BioLegend) for 20 min on ice. A minimum of 500,000 total cells were acquired and gated on the CD3^+^ cells. The CD8^+^ tetramer^+^ T-cell gate was set by matched no-tetramer stained isotype control (BioLegend). Compensation controls were checked regularly to avoid false-positive results and individually determined for each experimental setup.

### *Ex Vivo* IFN-γ Enzyme-Linked Immunospot (ELISPOT) Assay

Peripheral blood mononuclear cells of HLA-A*02^+^ HFRS patients were preserved in liquid nitrogen until use. Identification of the HTNV GP-derived CTL epitopes was performed using the Human IFN-γ precoated ELISPOT kit (DAKEWE Biotech Company, China). Briefly, a total of 2 × 10^5^ PBMCs in RPMI 1640 containing 10% FCS were seeded in each well of 96-well plates precoated with anti-human IFN-γ antibody, and stimulated with single peptide or peptide mixture (40 µmol/L). After 32 h of stimulation, the cells were removed, and then 100 µL biotinylated secondary antibody was added to each well and incubated at 37°C for 1 h. Next, the unbound secondary antibody was removed, and the samples were incubated with 100 µL/well streptavidin-HRP at 37°C for 1 h. Finally, the unbound streptavidin-HRP was removed, and 100 µL/well AEC solution was added to produce spots. Cells with 10 µg/mL phytohemagglutinin (DAKEWE Biotech Company, China) or no peptide stimulation were used as positive and negative controls, respectively. The spots representing epitope-specific IFN-γ-producing CTLs were counted using an automatic ELISPOT reader (Cellular Technology Limited, USA). Adjusted spot-forming cells (SFC) after subtracting negative values are expressed as SFC/10^6^ PBMCs.

### HLA-A2.1/K^b^ Tg Mice

HLA-A2.1/K^b^ Tg mice purchased from the Jackson Laboratory (Bar Harbor, ME, USA) were kindly provided by Dr. Yuzhang Wu (Third Military Medical University, Chongqing, China) and were raised in the Animal House Facility at the Center for Laboratory Animal, Fourth Military Medical University, Xi’an, China. The HLA-A2.1/K^b^ Tg mice created in a C57BL/6 background represent a chimeric gene consisting of the α1 and α2 domains of HLA-A*0201 and the α3 transmembrane and cytoplasmic domain of H-2K^b^. The HLA-A2.1/K^b^ Tg mice can be used as models to investigate peptide recognition *in vivo* restricted by human HLA-A*0201 molecules.

### Immunization of HLA-A2.1/K^b^ Tg Mice with Peptides

HLA-A2.1/K^b^ Tg mice were subdivided into six groups (*n* = 6 each). Immunizations of Tg mice with the HTNV GP nonapeptides were carried out using the N-terminal fragment of murine glycoprotein 96 (gp96) as an adjuvant (42). Briefly, 50 µg of HLA-A*0201-restricted peptides and 30 µg of the N-terminal fragment N333 (aa22–aa355) of murine gp96 were emulsified together in complete or incomplete Freund’s adjuvant (Difco) as mixture immunogen. Each 6- to 8-week-old male HLA-A2.1/K^b^ Tg mouse was immunized with the prepared mixture *via* subcutaneous injection at multiple sites. The injection volume was adjusted to 100 µL for each animal. Three immunization injections were administered to each group of mice at intervals of 10 days. The same method was used to immunize mice with the HLA-B*35-restricted epitope VY9 as the uncorrelated peptide control group. Subcutaneous injections with the commercial HFRS vaccine and PBS were used as the positive and negative control groups, respectively.

### HTNV Challenge

Ten days after the last immunization, the Tg mice were challenged with the HTNV 76-118 strain by intramuscular injection (1 × 10^5^ pfu/mouse). On the fourth day after HTNV challenge, the Tg mice were sacrificed. Tissue samples, including the cerebrum, heart, liver, spleen, lung, and kidneys of the Tg mice, were weighed and prepared as 10% (gram/milliliter) tissue suspensions in PBS. The tissue samples were then freeze-thawed (−80°C/37°C) three times after grinding and centrifuged at 250 × *g* for 30 min at 4°C to collect the supernatants.

### Detection of HTNV Antigens by Enzyme-Linked Immunosorbent Assay (ELISA)

Hantaan virus antigens in the supernatants of the tissue samples of Tg mice were detected by sandwich ELISA. The anti-HTNV NP mAb 1A8 was prepared in the Department of Microbiology of our university. The mAb 1A8 was used as the coating antibody, and the horseradish peroxidase-conjugated mAb 1A8 was used as the detecting antibody (17). The supernatants of normal tissue were used as negative controls. First, add 100 µL prepared supernatants to duplicate wells and incubate for 2 h at 37°C. Next, remove the supernatants and wash three times. Then, add 100 µL detecting antibody to each well and incubate for 1 h at 37°C. After that, remove the detecting antibody and wash three times. Add 100 µL TMB substrate to each well and incubate at room temperature. Finally, add 100 µL stop solution (0.16 M sulfuric acid) and measure the absorbance at 450 nm with a standard ELISA plate reader (Bio-red). Positive/negative (P/N) means the ratio of positive to negative absorbance value. An absorbance value exceeding 0.1- and the 2.1-fold of the negative control (P/N > 2.1) was considered positive and significant, which indicated that HTNV-specific antigen could be detected in the samples.

### Determination of Relative HTNV RNA Loads by Quantitative RT-PCR

The major target organs, including the liver, spleen, and kidneys of challenged Tg mice, were preserved in a non-frozen tissue RNA preservation solution (Solarbio, China) at −4°C. The tissue RNA of each organ was extracted using an RNAprep Pure Tissue Kit (Tiangen Biotech, China) and utilized as a template for reverse transcription to obtain cDNA with PrimeScript™ RT-PCR kit (Takara). The target RNA sequence of the HTNV S segment was detected in the major organs utilizing a SYBR real-time quantification PCR kit (Takara) with the following primers: HTNV forward, 5′-GATCAGTCACAGTCTAGTCA-3′; HTNV reverse, 5′-TGATTCTTCCACCATTTTGT-3′; mouse β-actin forward, 5′-AACAGTCCGCCTAGAAGCAC-3′; mouse β-actin reverse, 5′-CGTTGACATCCGTAAAGACC-3′. Each result was recorded as the cycle time (*C*_t_) and quantified by $2^{-\Delta \Delta C_{t}}$.

### Statistical Analysis

The statistical analysis was performed using SPSS 16.0 (SPSS Inc., Chicago, IL, USA) and GraphPad Prism software, version 6 (GraphPad; La Jolla, CA, USA). The Mann–Whitney *U* test and Student’s *t*-test were used for parameter comparison between two subject groups. The frequencies of the epitope-specific CTLs are presented as medians with corresponding interquartile ranges (IQRs). *p*-Values (two-tailed) below 0.05 (*p* ≤ 0.05) were considered to be statistically significant.

## Results

### Thirty-Four HTNV GP-Derived HLA-A*0201-Restricted Nonapeptides Were Predicted and Synthesized

Thirty-four HTNV GP-derived HLA-A*0201-restricted nonapeptides were predicted by utilizing the BIMAS and SYFPEITHI databases, ranked by their scores and synthesized at 90% purity. The sequences, locations and scores of 34 HTNV GP-derived HLA-A*0201-restricted nonapeptides are listed in Table 2.

**Table 2:** Information for the predicted and synthesized 34 Hantaan virus (HTNV) glycoprotein (GP)-derived HLA-A*0201-restricted nonapeptides.

No.	Sequence	Start aa	End aa	Bioinformatics and molecular analysis section		SYFPEITHI	
				Rank	Score	Rank	Score
1	VLIEGKCFV	179	187	1	2,666.276	5	27
2	VMASLVWPV	8	16	2	1,473.053	8	26
3	VLLSILCPV	1,121	1,129	3	1,006.209	7	27
4	FLLFSLVLL	1,115	1,123	4	836.253	3	28
5	SLFSLLPGV	458	466	5	591.888	2	30
6	SLTECPTFL	996	1,004	6	481.722	32	23
7	YIFTMWIFL	628	636	7	467.438	45	21
8	TMWIFLLVL	631	639	8	458.875	15	35
9	YQYETSWGC	757	765	9	227.995	–	–
10	VLIPAITFI	496	504	10	224.357	6	27
11	VLIVLCVFL	1,108	1,116	11	199.738	11	26
12	SVHIVCFFV	205	213	12	194.137	121	16
13	FLLVLESIL	635	643	13	149.071	22	24
14	LVLESILWA	637	645	14	141.813	60	19
15	IILTVLKFI	504	512	15	114.142	30	23
16	GIFSGNWIV	1,100	1,108	16	108.808	40	22
17	WIVLIVLCV	1,106	1,114	17	101.181	34	23
18	LIWTGMIDL	358	366	18	95.325	27	23
19	NMVSGYKKV	909	917	19	75.348	47	21
20	TLTRGQNTV	1,022	1,030	20	69.552	24	24
21	SLVWPVLTL	11	19	25	49.134	1	30
22	ALGPYRVQV	160	168	21	69.552	4	27
23	VLIEGKCFV	947	955	–	–	9	26
24	AAAPHLDKV	1,058	1,066	–	–	10	26
25	LIALGPYRV	158	166	47	16.258	12	25
26	LLPGVAHSI	462	470	45	17.736	13	25
27	AITFIILTV	500	508	35	21.996	14	25
28	FTLTCLVSL	989	997	–	–	16	25
29	SAPIYVPTL	253	261	–	–	17	24
30	GLFPKLNHT	341	349	23	63.417	18	24
31	VIGQCIYTI	448	456	–	–	19	24
32	YTITSLFSL	454	462	44	18.207	20	24
33	DLPGYYEAV	365	373	–	–	28	23
34	GLHAAAPHL	1,055	1,063	38	21.362	–	–

### Eight of 34 Predicted HTNV GP-Derived HLA-A*0201-Restricted Nonapeptides Exhibited High Binding Affinity to the HLA-A*0201 Molecules

**Figure 1:**
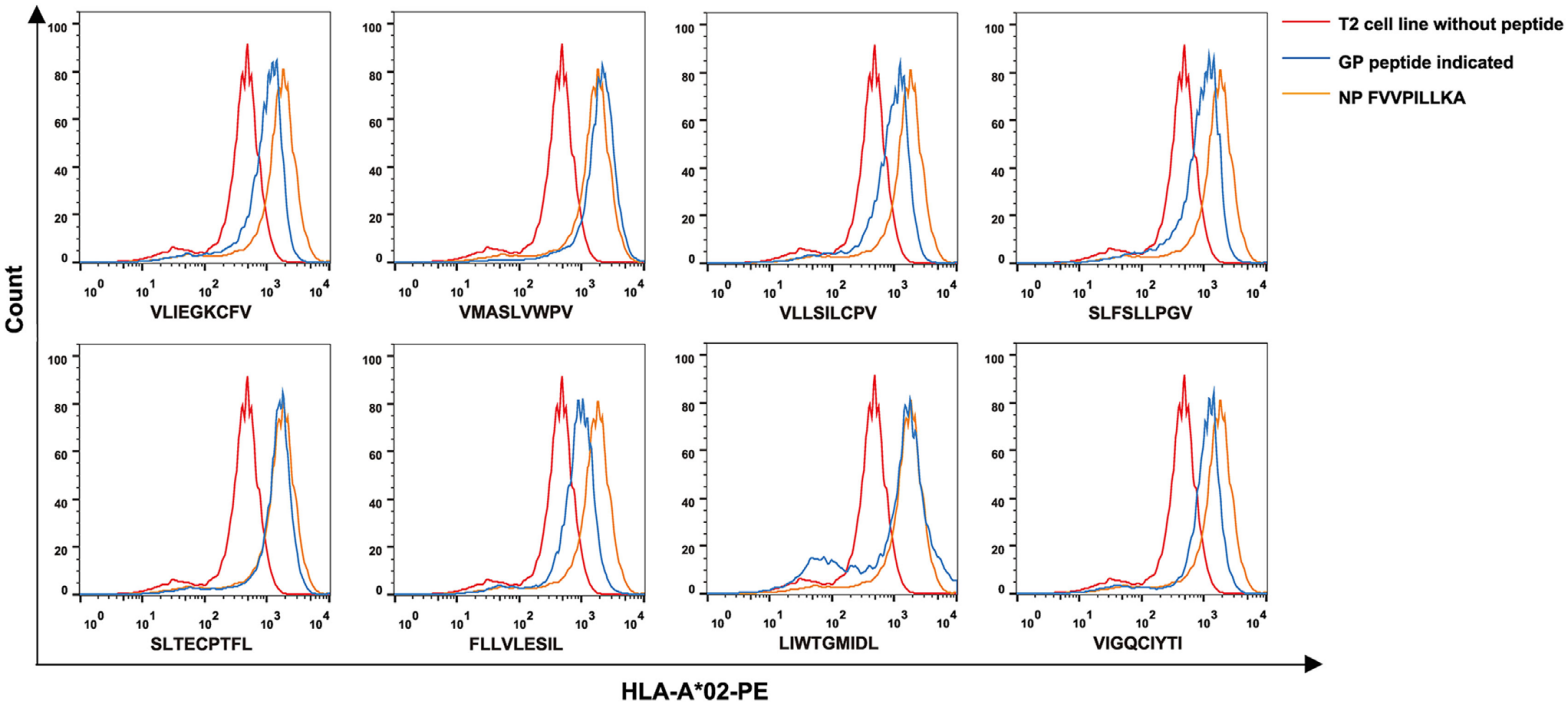
High binding affinity of eight Hantaan virus (HTNV) GP nonapeptides to HLA-A*0201 molecules. The T2 cell binding assay was used to quantify the peptide-binding affinity of 34 predicted HTNV GP nonapeptides to HLA-A*0201 molecules. T2 cells incubated with each peptide and β2-microglobulin were then stained with phycoerythrin-labeled HLA-A*02 monoclonal antibody and detected by flow cytometry. The orange curves indicate HLA-A*0201 stabilization with HLA-A*0201-restricted HTNV NP FA9 (aa129–aa137, FVVPILLKA) peptides serving as positive controls. The red curves indicate T2 cells incubated without peptide serving as negative controls. The blue curves indicate T2 cells incubated with each HTNV GP nonapeptide. The overlay of the three conditions in histograms clearly show that the curve of T2 cells incubated with each of the eight HTNV GP nonapeptides was shifted more to the right with a higher fluorescence intensity of HLA-A*0201 molecules than those incubated without peptide, indicating that these nonapeptides have a high binding affinity to the HLA-A*0201 molecule. GP, glycoprotein; NP, nucleoprotein.

To further investigate the binding affinity of nonapeptides to HLA-A*0201 molecules, the T2 cell binding assay was conducted. Eight of 34 predicted HLA-A*0201-restricted nonapeptides (VLIEGKCFV, VFV9; VMASLVWPV, VV9; VLLSILCPV, VPV9; SLFSLLPGV, SV9; SLTECPTFL, SL9; FLLVLESIL, FL9; LIWTGMIDL, LL9; VIGQCIYTI, VI9) increased the cell surface expression of HLA-A*0201 molecules and showed a high binding affinity to HLA-A*0201 molecules characterized by FI ≥ 1 (Figure 1). Notably, nonapeptides VV9, SL9, and LL9 with FI > 2 exhibited a higher binding affinity among them. The nonapeptide FA9 restricted by HLA-A*0201 was used as a positive control, and the T2 cell line without peptide was used as a negative control. Detailed information for the eight nonapeptides with high binding affinity is summarized in Table 3.

**Table 3:** Information for the eight screened Hantaan virus (HTNV) glycoprotein (GP)-derived HLA-A*0201-restricted high-binding affinity nonapeptides by the T2 cell binding assay.

HTNV structural protein	Sequence	Start aa	End aa	Bioinformatics and molecular analysis section		SYFPEITHI		T2 cell binding assay
				Rank	Score	Rank	Score	FI
GP	VLIEGKCFV	179	187	1	2,666.3	5	27	1.31
	VMASLVWPV	8	16	2	1,473.1	8	26	3.67
	VLLSILCPV	1,121	1,129	3	1,006.2	7	27	1.21
	SLFSLLPGV	458	466	5	591.9	2	30	1.23
	SLTECPTFL	996	1,004	6	481.7	32	23	2.35
	FLLVLESIL	635	643	13	149.1	22	24	1.13
	LIWTGMIDL	358	366	18	95.3	27	23	2.55
	VIGQCIYTI	448	456	71	5.609	19	24	1.50

NP (positive control)	FVVPILLKA	129	137	–	–	–	–	2.76

*FI, fluorescence index*.

*FI = [mean phycoerythrin (PE) fluorescence with the given peptide − mean PE fluorescence without peptide]/(mean PE fluorescence without peptide). FI ≥ 1 represents the high-affinity peptide*.

### Frequencies of Seven HLA-A*0201 High-Affinity HTNV GP Epitope-Specific CD8^+^ T Cells Were Associated with the Severity of HFRS Disease

**Figure 2:**
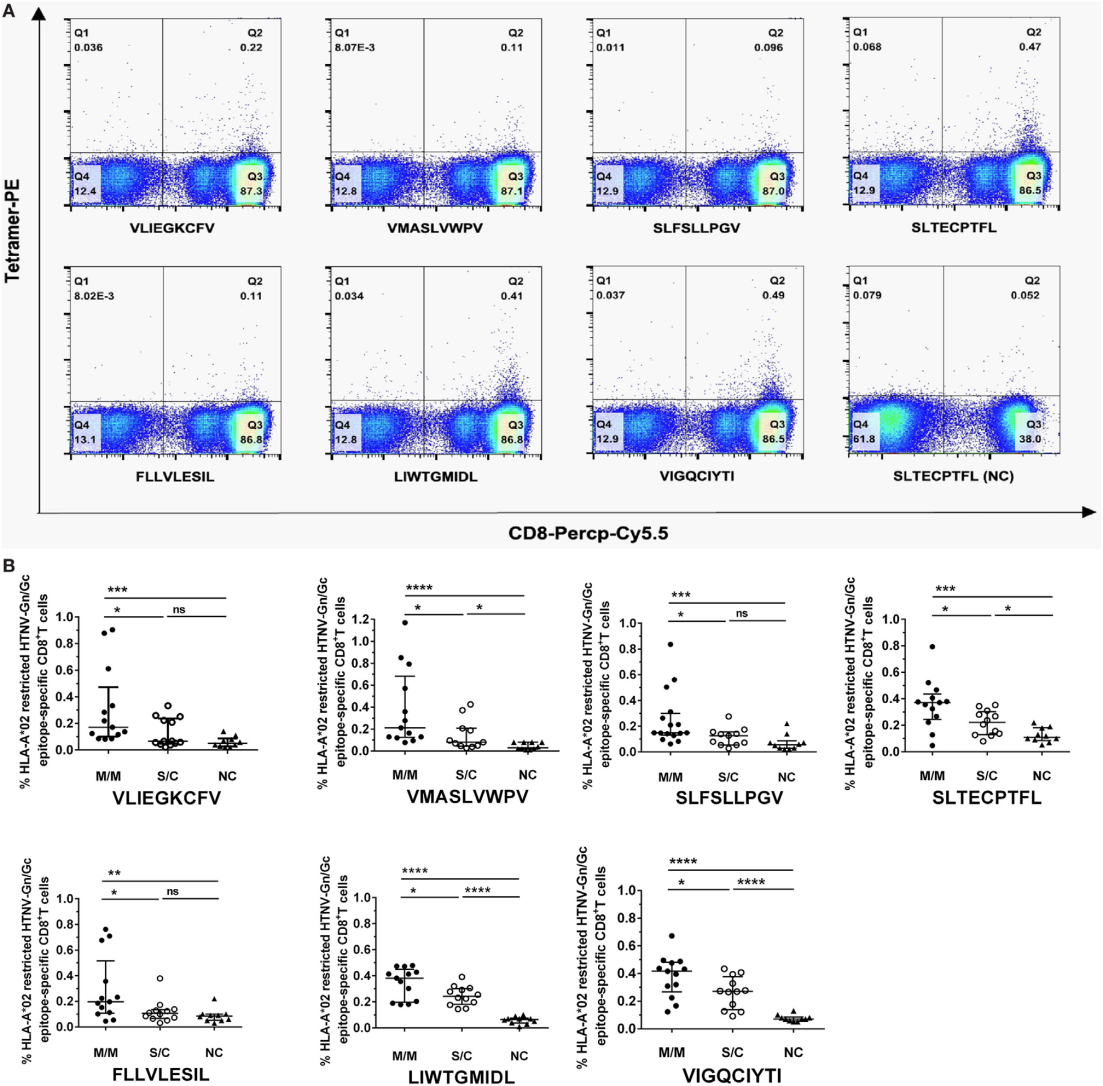
Frequencies of HLA-A*02-restricted Hantaan virus (HTNV) glycoprotein (GP) epitope-specific CTLs in peripheral blood mononuclear cells (PBMCs) of hemorrhagic fever with renal syndrome (HFRS) patients and their relationship with disease severity. The PBMCs from HLA-A*02^+^ HFRS patients in the acute phase of HFRS were stained with HLA-A*0201 tetramer preloaded with seven high HLA-A*0201-binding affinity peptides, respectively (including VLIEGKCFV, VMASLVWPV, SLFSLLPGV, SLTECPTFL, FLLVLESIL, LIWTGMIDL, and VIGQCIYTI). HLA-A*0201/peptide tetramer^+^ cells gated from CD3^+^CD8^+^ T lymphocytes were considered epitope-specific CTLs. **(A)** The frequencies of seven HTNV GP epitope-specific CTLs of the same HFRS patient (a 52-year-old male with moderate disease severity) and an HLA-A*02^+^ healthy donor [normal control (NC)]. **(B)** Comparison of the seven HLA-A*02-restricted HTNV GP epitope-specific CTL frequencies among patients with mild/moderate disease severity (M/M), patients with severe/critical disease severity (S/C) in the acute phase and NC. The Mann–Whitney *U* test was used to determine the significance of differences between two groups, and black lines represent the medians with the corresponding interquartile ranges. *p*-Values below 0.05 (*p* < 0.05) were considered statistically significant, *p < 0.05, **p < 0.01, ***p < 0.001, ****p < 0.0001, ns indicates no significant difference.

We focused on the eight HTNV GP nonapeptides that exhibited a high-binding affinity to the HLA-A*0201 molecules, and utilized them to synthesize peptide/HLA-A*0201 tetramer complexes to examine whether the eight nonapeptides could be recognized by the TCRs of CD8^+^ T cells in HLA-A*02^+^ patients with HFRS. Seven nonapeptides were successfully used to synthesize tetramers, whereas the nonapeptide VPV9 could not form a stable peptide/HLA-A*0201 tetramer complex. Thus, we used the seven peptide/HLA-A*0201 tetramers to determine whether the nonapeptides could be recognized by CD8^+^ T cells and quantify the frequencies of the nonapeptide-specific CD8^+^ T cells. HLA-A*02^+^ patients with a different severity of HFRS were tested at the acute phase during hospitalization. HLA-A*02^+^ subjects who were seronegative for HTNV served as NC. Epitope-specific tetramer^+^ CD8^+^ T cells could be detected in PBMCs, indicating that seven nonapeptides could be recognized by TCRs of CD8^+^ T cells (Figure 2A). The frequencies of HLA-A*0201-restricted epitope-specific CD8^+^ T cells in PBMCs are presented as medians with their corresponding IQRs. There were 0.17% (0.10–0.47%) for epitope VFV9, 0.21% (0.13–0.68%) for epitope VV9, 0.15% (0.13–0.30%) for epitope SV9, 0.37% (0.25–0.43%) for epitope SL9, 0.20% (0.11–0.52%) for epitope FL9, 0.38% (0.20–0.45%) for epitope LL9, and 0.42% (0.27–0.48%) for epitope VI9 in patients with mild/moderate disease; 0.07% (0.05–0.24%) for epitope VFV9, 0.08% (0.05–0.21%) for epitope VV9, 0.13% (0.05–0.16%) for epitope SV9, 0.22% (0.13–0.30%) for epitope SL9, 0.11% (0.07–0.14%) for epitope FL9, 0.24% (0.18–0.30%) for epitope LL9, and 0.27% (0.14–0.38%) for epitope VI9 in patients with severe/critical disease; and 0.05% (0.03–0.09%) for epitope VFV9, 0.03% (0.02–0.08%) for epitope VV9, 0.06% (0.03–0.09%) for epitope SV9, 0.11% (0.09–0.19%) for epitope SL9, 0.09% (0.05–0.10%) for epitope FL9, 0.07% (0.04–0.08%) for epitope LL9, and 0.07% (0.06–0.09%) for epitope VI9 in NC. Notably, the frequencies of seven HTNV GP nonapeptide-specific CD8^+^ T cells in the patients with mild/moderate disease were significantly higher than these in the patients with severe/critical disease and NC (*p* < 0.05, Figure 2B), indicating that the seven HTNV GP-derived epitopes might induce protective CTL responses in HLA-A*02^+^ HFRS patients. In addition, epitopes VV9-, SL9-, LL9-, and VI9-specific CD8^+^ T cells revealed higher frequencies compared with other epitope-specific CD8^+^ T cells in patients with mild/moderate disease and showed significantly higher frequencies in patients with severe/critical disease than these in NC (*p* < 0.05, Figure 2B), while no significant difference was found between VFV9-, SV-, and FL-specific CD8^+^ T cells of patients with severe/critical disease and NC. This may due to the better capability of epitope VV9, SL9, LL9, and VI9 to stimulate proliferation of epitope-specific CD8^+^ T cells.

### Seven HTNV GP Nonapeptides Could Elicit Epitope-Specific CD8^+^ T-Cell Responses in HLA-A*02^+^ HFRS Patients

**Figure 3:**
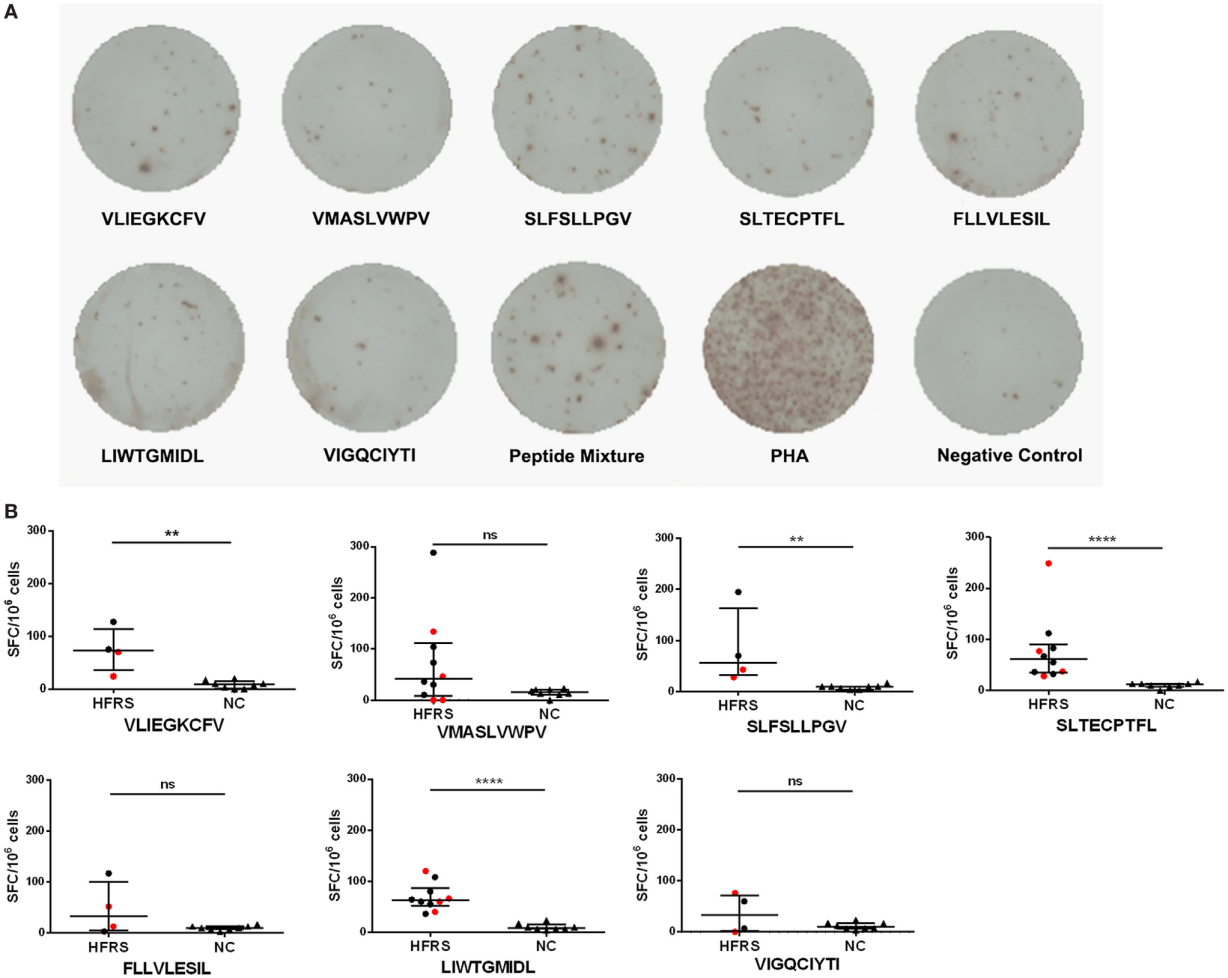
Detection of Hantaan virus (HTNV) glycoprotein (GP) epitope-specific CTL responses in patients with hemorrhagic fever with renal syndrome (HFRS). An *ex vivo* IFN-γ enzyme-linked immunospot assay was used to test the epitope-specific IFN-γ-producing CTLs of patients with HFRS. **(A)** The peripheral blood mononuclear cells of HLA-A*02^+^ patients with HFRS (a 47-year-old male with moderate disease severity) were stimulated with 40 µmol/L single peptide or peptide mixture or 10 µg/mL phytohemagglutinin (PHA) *in vitro*. The notes under the wells indicate the sequences of peptides or the name of the stimulant. Cells stimulated with PHA served the positive control, and cells with no stimulation were the negative control. **(B)** Comparison of the numbers of spot-forming cells (SFC) between HFRS patients and HLA-A*02^+^ healthy donors [normal controls (NC)]. The black dots represent patients with mild/moderate disease severity and the red dots represent patients with severe/critical disease severity. The numbers of epitope-specific INF-γ-secreting SFC were calculated by subtracting the numbers of spots in the negative controls from those in the stimulated samples. The Mann–Whitney *U* test was used to determine the significance of differences between two groups, and black lines represent the medians with the corresponding interquartile ranges. *p*-Values below 0.05 (*p* < 0.05) were considered statistically significant, **p < 0.01, ****p < 0.0001, ns indicates no significant difference.

Having demonstrated that seven nonapeptides could bind to HLA-A*0201 molecules and were recognized by TCRs to form MHC-peptide-TCR complexes, we next assessed whether these nonapeptides could elicit epitope-specific T-cell responses in patients with HFRS. Thus, we used single peptide or a peptide mixture (including the seven nonapeptides) to stimulate PBMCs of HLA-A*02^+^ patients with HFRS, and detected the frequencies of IFN-γ-producing T cells by ELISPOT assay. Notably, the results showed that either the peptide mixture or single peptide could induce IFN-γ production from the PBMCs of HFRS patients *in vitro* (Figure 3A), indicating that each of the seven HTNV GP epitopes could effectively elicit epitope-specific IFN-γ-secreting T-cell responses. Next, we analyzed the positive responses at the single-peptide level. For the limited ELISPOT data of each peptide, we could not perform the statistical analysis between different severity groups. Here, we showed the numbers of epitope-specific CTLs and make a comparison between HFRS patients and NC. The numbers of epitope-specific CTLs are presented as medians with their IQRs. There were 73 (35–114) SFC/10^6^ PBMCs for epitope VFV9, 41 (8–112) SFC/10^6^ PBMCs for epitope VV9, 57 (32–164) SFC/10^6^ PBMCs for epitope SV9, 61 (35–90) SFC/10^6^ PBMCs for epitope SL9, 33 (6–101) SFC/10^6^ PBMCs for epitope FL9, 62 (51–87) SFC/10^6^ PBMCs for epitope LL9 and 33 (2–72) SFC/10^6^ PBMCs for epitope VI9 in HFRS patients, and 9 (1–15) SFC/10^6^ PBMCs for epitope VFV9, 15 (11–20) SFC/10^6^ PBMCs for epitope VV9, 10 (4–10) SFC/10^6^ PBMCs for epitope SV9, 12 (8–13) SFC/10^6^ PBMCs for epitope SL9, 10 (7–13) SFC/10^6^ PBMCs for epitope FL9, 9 (7–15) SFC/10^6^ PBMCs for epitope LL9, and 10 (7–17) SFC/10^6^ PBMCs for epitope VI9 in NC. The numbers of epitopes VFV9-, SV9-, SL9-, and LL9-specific CD8^+^ T cells were larger in HFRS patients than these in NC (*p* < 0.05, Figure 3B). Notably, when remove the data of patients with severe/critical disease (red dots), the numbers of epitope VV9-specific CD8^+^ T cells were also significantly larger than that in NC (*p* < 0.05, Figure 3B), indicating patients with milder disease may have higher VV9-specific CD8^+^ T-cell responses. The median numbers of epitope-specific IFN-γ-secreting CTLs in different disease severity group also showed the same tendency that patients with milder disease severity had more epitope-specific IFN-γ-secreting CTLs than these in patients with severer disease severity (Table S1 in Supplementary Material).

### Immunization with the HLA-A*02-Restricted HTNV GP Epitopes Could Reduce HTNV Titers in Tissue Supernatants of HLA-A2.1/K^b^ Tg Mice after HTNV Challenge

Since we found that higher frequencies of epitope-specific CD8^+^ T cells were associated with milder disease severity, we next conducted HTNV challenge experiments to determine whether the epitope-specific CD8^+^ T-cell responses could provide protection against HTNV infection *in vivo*. To achieve this goal, we used three of the seven epitopes, VV9, SL9, and LL9, which showed higher HLA-A*0201-binding affinity and higher frequencies of epitope-specific CD8^+^ T cells in patients with mild/moderate severity, to immunize the HLA-A2.1/K^b^ Tg mice. Subsequently, we established a model of HTNV infection based on replication of the virus *in vivo*.

**Figure 4:**
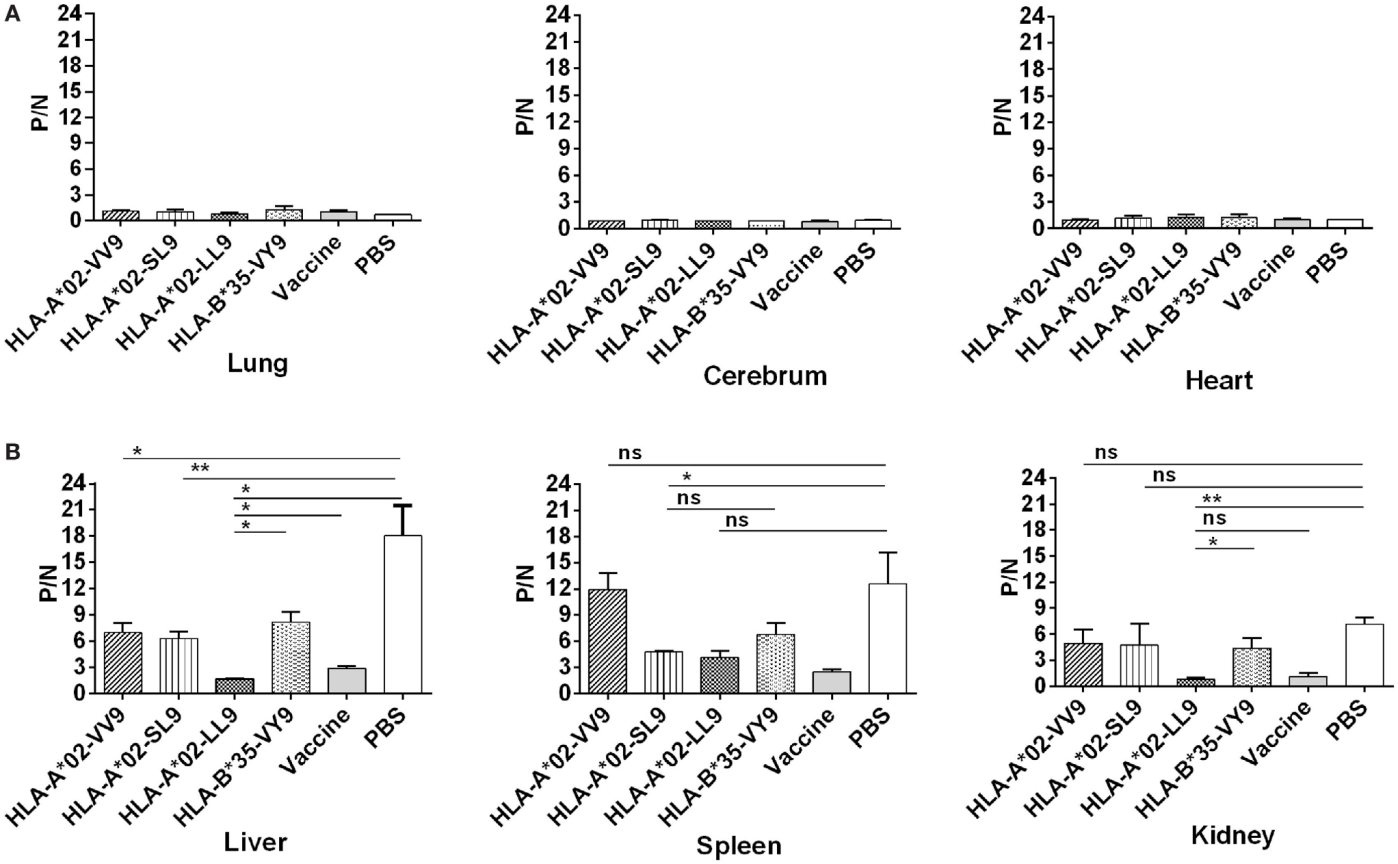
Detection of Hantaan virus (HTNV) antigen by enzyme-linked immunosorbent assay (ELISA) in the major organs of HLA -A2.1/K^b^ transgenic mice (Tg) after HTNV challenge. The HLA-A2.1/K^b^ Tg mice were divided into six groups (*n* = 6 each) according to the different immunizations, including mice immunized with the HLA-A*02-restricted HTNV GP epitopes VV9 (VMASLVWPV), SL9 (SLTECPTFL), and LL9 (LIWTGMIDL), the HLA-B*35-restricted HTNV NP epitope VY9 (VPILLKALY), the hemorrhagic fever with renal syndrome (HFRS)-inactivated vaccine or PBS. The group immunized with the HLA-B*35-restricted HTNV NP epitope VY9 (unrelated peptide) and PBS served as negative controls. The group immunized with the HFRS vaccine served as the positive control. Ten days after the final immunization booster, the mice were challenged with the HTNV 76-118 strain and sacrificed 4 days after HTNV challenge. The HTNV antigen (*y*-axis) levels were detected in tissue supernatants of the lung, liver, cerebrum, spleen, kidneys, and heart (*x*-axis) of HLA-A2.1/K^b^ Tg mice after HTNV infection in the six groups. **(A)** Few or no detectable HTNV antigen was detected in the lung, cerebrum, and heart in any of the six groups of immunized Tg mice after HTNV challenge by ELISA. **(B)** Comparison of the HTNV antigen levels among six immunization groups in the tissue supernatants of liver, spleen, and kidneys, respectively. Positive/negative (P/N) means the ratio of positive to negative absorbance value. An absorbance value exceeding 0.1- and the 2.1-fold of the negative control (P/N > 2.1) was considered positive and significant, indicating that the HTNV-specific antigen could be detected in the sample. The Student’s *t*-test was used for statistical evaluation. *p*-Values below 0.05 (*p* < 0.05) were considered statistically significant. *p < 0.05, **p < 0.01, ns indicates no significant difference.

In our previous study, we detected HTNV titers in six organs of the HTNV-infected naïve HLA-A2.1/K^b^ Tg mice, and found that high levels of HTNV antigens could be detected in the liver, spleen, and kidneys, but not in the lungs, cerebrum, and heart of the Tg mice, suggesting that the liver, spleen, and kidneys are the major organs for infection and replication of HTNV in HLA-A2.1/K^b^ Tg mice (6). Consistent with the previous results, few or no detectable virus was found in the lung, cerebrum, and hearts in any of the six groups of immunized Tg mice after HTNV challenge based on the detection of HTNV antigens in the tissue supernatants by ELISA (Figure 4A).

After HTNV challenge, each Tg mouse from the negative control group injected with PBS showed evidence of high-titer HTNV antigen in the tissue supernatants of the liver, spleen, and kidney, whereas different reduced levels of the mean viral titer were observed in the organs of the mice immunized with the HFRS vaccine and each epitope (Figure 4B). Compared with the negative control Tg mice, immunization with epitope VV9 led to a significant decrease in mean HTNV titers in the supernatants of the livers (*p* < 0.05, Figure 4B), immunization with epitope SL9 led to a significant decrease in the liver and spleens (*p* < 0.01 for liver and *p* < 0.05 for spleen, Figure 4B), and immunization with epitope LL9 led to a significant decrease in the liver and kidneys (*p* < 0.05 for liver and *p* < 0.01 for kidney, Figure 4B). Importantly, epitope LL9-immunized mice showed significantly lower levels of HTNV titers in the liver and kidneys in comparison to the irrelevant control peptide VY9-immunized Tg mice (*p* < 0.05 for liver and *p* < 0.01 for kidney, Figure 4B), and similar levels in comparison to the HFRS vaccine-immunized mice (Figure 4B), which suggested that CD8^+^ T-cell responses induced by epitope LL9 could strongly inhibit virus replication and clear the HTNV in the liver and kidneys.

### Immunization with the HTNV GP Epitopes Could Inhibit HTNV Replication in HLA-A2.1/K^b^ Tg Mice to Protect against HTNV Challenge

**Figure 5:**
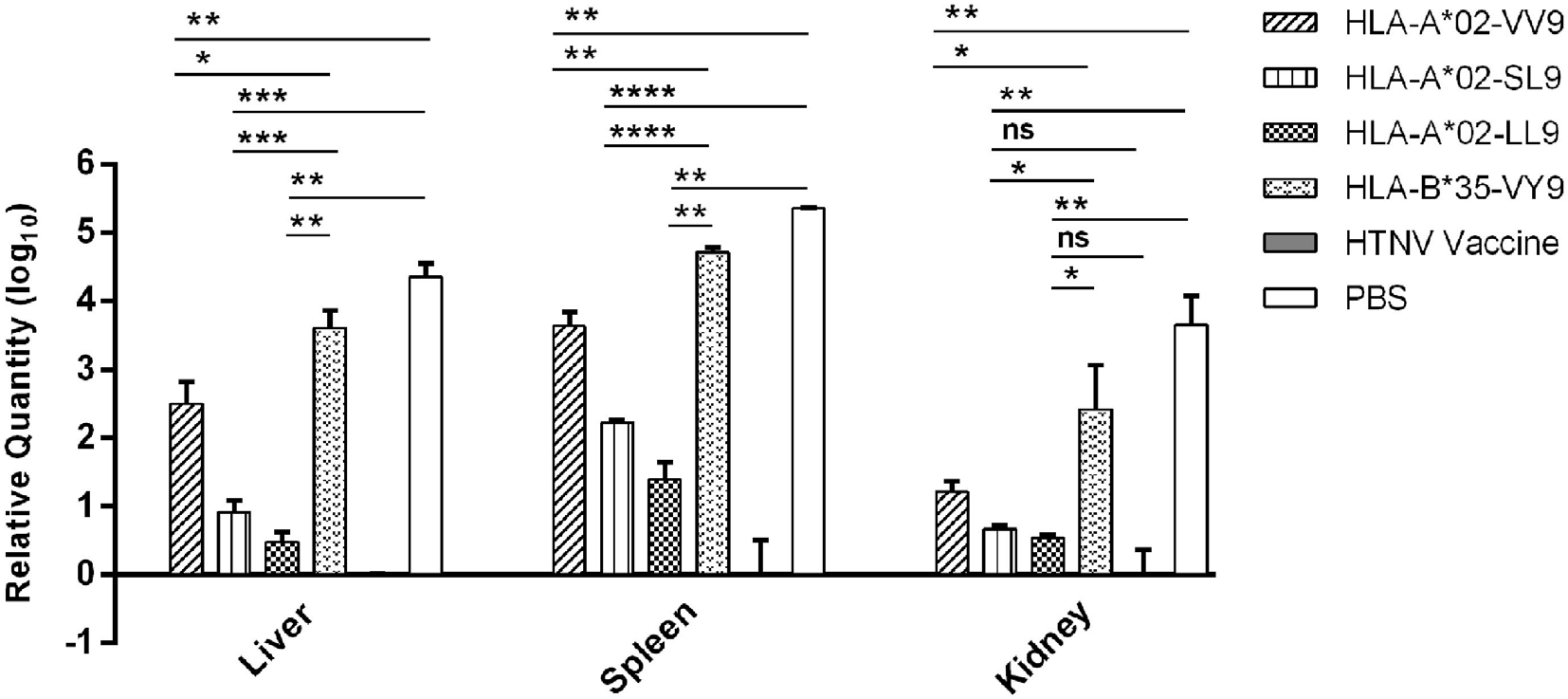
Detection of Hantaan virus (HTNV) RNA loads *via* RT-PCR in the major organs of HTNV-challenged HLA-A2.1/K^b^ transgenic (Tg) mice. The HLA-A2.1/K^b^ Tg mice were divided into six groups (*n* = 6 each) according to the different immunizations, including the mice immunized with the HLA-A*02-restricted HTNV glycoprotein epitopes VV9 (VMASLVWPV), SL9 (SLTECPTFL), and LL9 (LIWTGMIDL), the HLA-B*35-restricted HTNV NP epitope VY9 (aa131–aa139, VPILLKALY), the hemorrhagic fever with renal syndrome (HFRS)-inactivated vaccine or PBS. The group immunized with the HLA-B*35-restricted HTNV NP epitope VY9 (unrelated peptide) and PBS served as negative controls. The group immunized with the HFRS vaccine served as the positive control. Ten days after the final immunization booster, the Tg mice were challenged with the HTNV 76-118 strain and sacrificed 4 days after HTNV challenge. The HTNV RNA loads (*y*-axis) were detected in the liver, spleen, and kidneys of each immunized group (*x*-axis) after HTNV challenge by RT-PCR. The results shown were comparison of the HTNV RNA loads among six immunization groups and recorded as the cycle time (*C*_t_) and quantified by $2^{-\Delta \Delta C_{t}}$. The Student’s *t*-test was used for statistical evaluation. *p*-Values below 0.05 (*p* < 0.05) were considered statistically significant. *p < 0.05, **p < 0.01, ***p < 0.001, ****p < 0.0001, ns indicates no significant difference.

Previous results indicated that liver, spleen, and kidneys of HLA-A2.1/K^b^ Tg mice were target organs of HTNV, and immunization with HTNV GP epitopes VV9, SL9, and LL9 could decrease HTNV antigen levels in tissue supernatants of HTNV-infected Tg mice. For further protective effect evaluation of HTNV GP epitopes, we used a more sensitive and accurate assay, real-time PCR, to determine the HTNV S segment RNA loads in the target organs of epitopes VV9, SL9, and LL9-immunized Tg mice after challenge with HTNV. In accordance with the HTNV antigen detection, Tg mice injected with PBS or immunized with the control peptide VY9 showed high HTNV RNA loads in the target organs, and few or no detectable HTNV RNA loads were observed in the target organs of the mice immunized with the HFRS vaccine (Figure 5).

Notably, we detected significant differences in the HTNV RNA loads between the Tg mice immunized with HTNV GP epitopes and the mice injected with PBS or immunized with the control peptide VY9. The HTNV RNA loads in the liver, spleen, and kidneys of mice immunized with epitopes VV9, SL9, and LL9 were all significantly lower than the levels in mice injected with PBS or immunized with the control peptide VY9 (*p* < 0.05, Figure 5), indicating that VV9, SL9, and LL9 epitope-specific CD8^+^ T-cell responses could inhibit HTNV replication in the liver, spleen, and kidneys of Tg mice. HFRS inactivated vaccine-immunized Tg mice showed lower HTNV RNA loads in liver and spleen compared with epitope VV9, SL9, or LL9-immunized Tg mice (*p* < 0.05, Figure 5). Interestingly, there was no significant difference in kidneys of HTNV vaccine-immunized Tg mice compared with the HTNV RNA loads of SL9 or LL9-immunized Tg mice (*p* > 0.05, Figure 5), suggesting that epitopes SL9 and LL9 might induce preponderant CD8^+^ T-cell responses in kidneys to inhibit virus replication.

Since epitopes VV9, SL9, and LL9 all could induce CD8^+^ T-cell responses to inhibit HTNV replication, we further compared the HTNV RNA loads among VV9, SL9, and LL9-immunized Tg mice to explore which epitope had the strongest protective effect in different target organs. In the liver, spleen, and kidneys, both SL9 and LL9-immunized Tg mice had significantly lower HTNV RNA loads compared with VV9-immunized Tg mice (*p* < 0.05, Figure 5), suggesting that SL9- and LL9-induced CD8^+^ T-cell responses had a stronger effect on inhibiting HTNV replication than VV9-induced CD8^+^ T-cell responses. Lower HTNV RNA loads were observed in LL9-immunized Tg mice than in SL9-immunized Tg mice (Figure 5), indicating that LL9-induced CD8^+^ T-cell responses might play a more critical role against HTNV.

## Discussion

We previously reported eight HTNV NP-derived CTL epitopes and demonstrated that the HLA-A*02-restricted CTL epitope FVVPILLKA on NP could display polyfunctional activities and mediate effective protective responses in HLA-A2.1/K^b^ Tg mice (6, 20, 30). In this study, we first identified seven HTNV GP-derived HLA-A*0201-restricted CTL epitopes based on their high binding affinity to HLA-A*0201 molecules, ability to bind to TCRs and ability to elicit IFN-γ-producing CTL responses. Importantly, the frequencies of seven epitope-specific CTLs were higher in patients with mild/moderate severity than in those with severe/critical severity. Notably, epitopes VV9, SL9, and LL9 showed higher binding affinity to HLA-A*0201 molecules and frequencies of epitope-specific CTLs in PBMCs of HFRS patients. Further evidence indicated that immunization with epitopes VV9, SL9, and LL9 successfully generated protective CTL responses to inhibit HTNV infection after HTNV challenge in HLA-A2.1/K^b^ Tg mice. Especially, immunization with epitope LL9 dramatically inhibited HTNV replication in all target organs (liver, spleen, and kidneys) of Tg mice, which suggested that epitope LL9 might be a good candidate for use in the design of effective HTNV peptide vaccines against HTNV.

Vaccination is the most effective means for preventing viral infection. However, individuals immunized with HFRS vaccine may still develop HFRS disease due to limited neutralizing antibodies secreting or cellular immune responses (15–17). Thus, the development of more effective HTNV vaccines is necessary. Emerging results have revealed that vaccines that strictly elicit immunodominant epitope-specific CTL responses could be more effective against viruses. Vaccines that induce both immunodominant and subdominant epitope responses were significantly less protective than vaccines that only elicited immunodominant epitope-specific responses, suggesting that the increased breadth of T-cell epitope recognition may prevent the induction of optimal protective CTL immunity and reduce the efficiency of host immunity against pathogens (43, 44). The secondary CTL expansion in vaccinated mice competes for antigen accessibility on antigen-presenting cells, suppressing the priming of other protective pathogen-specific CTLs (45) and potentially explaining how subdominant epitope vaccination could reduce immunodominant epitope-induced CTL responses. Since HFRS vaccines have the potential to elicit both immunodominant and subdominant epitope-specific CTL responses, immunization with the inactivated vaccines may restrict the induction of optimal CTL responses. Notably, peptide vaccines are available for targeted prevention by eliciting epitope-specific CTL responses. The identification of protective immunodominant epitopes from HTNV may be feasible for the design of safer and more effective vaccines against HTNV infection. The Chinese Han population has developed stable gene polymorphisms of the HLA -A, -B and -DRB1 loci over a long evolutionary process in which HLA-A*02 alleles predominate as much as 29.7% (35, 36). Thus, we focused on HLA-A*02-restricted immunodominant epitopes.

Here, we demonstrated that seven HTNV GP-derived HLA-A*0201-restricted epitopes all could be recognized by the TCRs of CTLs in PBMCs of HFRS patients, indicating that these epitopes presented on HLA-A*02^+^ HTNV-infected cells may function as targets of specific CTLs. Interestingly, our results showed that patients with mild/moderate HFRS severity during the acute phase had higher frequencies of HTNV GP epitope-specific CTLs than patients with severe/critical severity, suggesting that these epitope-specific CTLs might play protective roles to reduce the severity of HFRS at early time points by recognizing the epitopes presented on HTNV-infected cells. The tetramer^+^ results of 0.1% or less, which were approximate to the frequencies of NC, were mainly in patients with severe/critical HFRS, also suggesting that patients with lower frequencies of HTNV GP-specific CTLs during the early time of HTNV infection may develop severer HFRS. These results are consistent with our previous studies showing that increased and vigorous HTNV NP-specific CTL responses correlated with milder HFRS (19, 20). Other studies also indicated the protective effect of CTL responses. In a hamster model, activation of T-cell immune responses was not responsible for the immunopathogenesis of HPS-like disease, but perhaps the innate immune responses, either elicited from infected endothelial cells, macrophages or neutrophils, contributed to the disease (46). In a mouse model of persistent infection, almost no IFN-γ-producing T cells were detected, while mice with transient infection showed increased functional HTNV-specific CTLs (23, 47). HTNV-specific T-cell responses in patients with severe HFRS were heavily impaired at the early stage of infection, indicating that an initial failure to mount strong T-cell responses may be the primary cause of the progression of acute HTNV infection into severe HFRS (27). In our study, when remove the ELISPOT data of patients with severe/critical disease, the numbers of epitope VV9-specific CD8^+^ T cells were significantly larger than that in NC, indicating patients with milder disease may have more functional epitope-specific CD8^+^ T cells. Thus, functional HTNV epitope-specific CTLs at early time points of HTNV infection are important for HTNV clearance and prevention of HFRS development. We demonstrated that the seven HTNV GP nonapeptides were able to induce IFN-γ-producing CTL responses. Considering that patients with milder severity had higher frequencies of epitope-specific CTLs during the acute phase of infection, we speculated that HTNV GP epitope-specific CTLs might have been generated at an early time point after HTNV infection and elicited effective protective IFN-γ-producing CTL responses against HTNV in patients with milder HFRS rather than in those with more severe infection.

However, Kilpatrick et al. identified three HLA-B*3501-restricted CTL epitopes in SNV and found significantly higher frequencies of the three epitope-specific CTLs in patients with severe HPS than in those with moderate HPS (32). There are some important factors that influence the balance between protective CTL responses that contribute to viral clearance and intense activation of CTLs that may trigger immunologic injury. Different HLA allele restrictions and virus species may be the considerable factors that impact CTL responses and disease outcome, which should be taken into account in studies of CTL responses against virus infection. Notably, in Kilpatrick’s study, the total frequencies of three SNV epitope-specific CTLs reached 44.2% in patients with severe HPS, while those in patients with moderate HPS did not exceed 10% (32). In the present study, the frequencies of each HTNV GP epitope-specific CTL maintained a relatively lower range (0.02–1.17%). Our previous study also showed that HTNV NP epitope FA9-specific CTLs, which also contributed to milder HFRS, maintained a lower frequency (0.01–0.70%). Hence, the frequencies of activated specific CTLs maintained in an optimum range may be an appropriate way to elicit effective responses against virus while avoiding damage to the host.

Notably, in our study, the frequencies of seven epitope-specific CTLs tested by ELISPOT assay all were lower than these tested by tetramer staining. Tuuminen et al. also found that PUUV NP epitope-specific CTLs measured by IFN-γ ELISPOT were lower in acute PUUV infection, indicating that increased epitope-specific CTLs which can be visualized by direct tetramer staining may not readily detectable by an *in vitro* functional assay such as IFN-γ ELSIPOT (48). Both tetramer staining and ELISPOT assay met validation criteria, while the tetramer staining showed better precision (49, 50). Tetramer staining mimics the molecular interaction between peptide-HLA complexes and TCRs that initiate T-cell activation *in vitro*. ELISPOT assay relies on a functional readout of cytokine secretion *in situ* to enumerate responding T cells (51). The overwhelming amount of antigen and different thresholds of peptide stimulation may drive the T cells into apoptosis by activation-induced cell death and lead clonal exhaustion (52, 53). Exhausted and terminally differentiated cells could die during lengthy incubation (24–48 h) before producing enough IFN-γ to be measured by ELISPOT assay. As to tetramer staining, the unresponsive cells could be measured, which may explain why the frequencies of epitope-specific CTLs tested by ELISPOT were lower (54). CTLs in a dysfunctional state could be restored after incubation with IL-2, suggesting that cytokines produced by Th cells may maintain the function of the CTLs (55), However, antigen-specific CTLs may lack effector activity *in vitro* for loss of other stimulation *in vivo* and could not be detected by ELISPOT, which could also contribute to the differences between tetramer staining and ELISPOT assay. Hobeika et al. set the mean + 2 SDs of response of CMV seronegative subjects as cutoff values and found that both tetramer staining and ELISPOT assay had a high sensitivity of 87.5% and specificity of 95–100% (56). In our study, receiver operating characteristic (ROC) curve analysis was used to determine the cutoff value for a positive versus negative tetramer score. When set the optimal operating point, which showed the largest positive likelihood ratio, as the cutoff value of each tetramer staining, the sensitivity and specificity ranged from 57.7 to 100% and from 60 to 90% (Table S2 in Supplementary Material). Among them, LL9/HLA-A*0201 tetramer staining had a high sensitivity of 100% and specificity of 90% with the cutoff value 0.09%, which may have the potential to be applied to clinical assistant diagnostic of HTNV infection.

Having demonstrated that seven HLA-A*02-restricted HTNV GP nonapeptide-specific CTL epitopes had immunoreactivity *in vitro*, we next conducted HTNV challenge experiments to determine whether these nonapeptides had immunogenicity that could elicit effective CTL responses against HTNV infection *in vivo*. Humanized mice, which are mice engrafted with human tissue and/or engineered to express human genes, can mimic human immunity against species-restricted pathogens *in vivo*. The humanized HLA-A2.1/K^b^ Tg mice, which express a chimeric gene consisting of the α1 and α2 domains of HLA-A*0201 molecule and the α3 transmembrane and cytoplasmic domain of H-2K^b^, can be a powerful tool to establish an HTNV replication model and explore the effect of immune responses primed by HTNV GP-derived HLA-A*0201-restricted epitopes *in vivo* (6, 57). Here, vaccination with HLA-A*0201-restricted HTNV GP epitopes VV9, SL9 and LL9 significantly reduced HTNV antigen levels and RNA loads in target organs of HLA-A2.1/K^b^ Tg mice compared with Tg mice injected with PBS or immunized with the control peptide VY9 after HTNV challenge, indicating that three HTNV GP epitopes all could induce protective CTL responses to inhibit HTNV replication and might elicit similar effects in humans. Although the proteomes of pathogens contain abundant peptides that have the potential to act as MHC I-restricted epitopes, CTL responses tend to be focused on some limited dominant epitopes, which are considered to be good candidates for vaccines (58). LL9-immunized Tg mice had lower HTNV antigens and RNA loads levels compared with LL9- and SL9-immunized Tg mice and even showed equivalent levels in kidneys compared with HTNV vaccine-immunized mice, suggesting that LL9 might function as an immunodominant epitope to induce optimal CTL responses against HTNV infection.

Previous studies indicated that Hantavirus Gn-derived epitopes were immunodominant compared with those from NP and Gc and were considered protective antigens to represent major candidates for genetically engineered vaccines (17, 33, 34). In our study, the immunodominant epitope LL9 (aa358–aa366) also derives from Gn (aa19–aa648), while the subdominant epitopes VV9 (aa8–aa16) and SL9 (aa996–aa1004) are located in a region of the signal peptide (aa1–aa18) and Gc (aa649–aa1135), respectively. Thus, the immunodominance of Gn is an interesting finding. Hantaviruses contain conserved tyrosine residues near the C-terminus of Gn that form an immunoreceptor tyrosine activation motif (ITAM) (aa611–aa634 for HTNV), which could promote polyubiquitination of the Gn polypeptide to facilitate its degradation *via* the proteasome (46, 59). The proteasome generates the epitopes presented on MHC I molecules that elicit CTL responses (60). After hantavirus infection, increased Gn epitopes produced by enhanced ITAM-regulated degradation are presented on the cell surface with MHC I molecules, and then the epitopes are recognized by specific CTLs and induce high-frequency CTL responses against target cells, which might explain the more effective Gn-related vaccine-induced CTL inhibition of virus replication after virus challenge *in vivo*.

The peptides presented by MHC class I molecules are generally restricted to 8–10 amino acids in length, and the peptides that bind to the same MHC I allele share a similar “peptide-motif,” which contains certain anchor residues that are preferentially utilized as anchors with MHC I molecules. The specific consensus peptide motif for HLA-A*0201 is a 9-mer peptide with dominant anchor residues of leucine (L) at position 2 and valine (V) or leucine (L) at the C-terminus (39). Interestingly, the LL9 sequence LIWTGMIDL is relatively conserved in SEOV (LIWRGLIDL) and DOBV (LIWEGYIDL) and is characterized by invariable primary anchor residues and few substitutions of the internal sequence. Although the internal sequence contexts of individual peptides would affect the binding affinity to MHC I molecules (either increase or decrease the binding affinity), substitutions of the secondary anchor amino acids at positions (3, 6, and 7) seems to have a limited effect on the immunogenic properties of peptides (39). More encouragingly, it has been demonstrated that HLA-A*0201-restricted peptides derived from influenza A virus proteins could elicit broad protection against distinct strains based on the induced-fit molecular mimicry of the TCR effectively targeting mutated peptides (61). Hence, it is possible that LL9 may induce CTL responses against SEOV and DOBV, which are also major causative agents of HFRS (3, 4).

Epitopes with professional characteristics lay a foundation for the development of more effective preventative or therapeutic strategies. Mixtures of synthetic peptides derived from cat allergen epitopes have been successfully used to induce tolerance in clinical phase 2 tests (62). A peptide vaccine targeting angiotensin II was recently developed as a novel treatment for hypertension and the prevention of heart failure (63). Applications of immunodominant-epitope-based cancer vaccines combined with other strategies have significantly enhanced the specificity and safety of T-cell responses targeting to tumors (64–68). HLA-restricted epitopes used to induce epitope-specific CTL responses that eliminate infected cells represent a promising vaccine strategy against dengue and influenza virus infection (69, 70). Identification of the HTNV GP epitope LL9 that can induce protective immune responses against HTNV infection in HLA-A2.1/K^b^ Tg mice may promote the development of a novel HTNV vaccine or related therapeutic methods for HFRS. Notably, the human kidney, as the major target organ of HTNV, is frequently injured after HTNV infection, and the injury may progress to lethal AKI (5). Higher levels of HTNV-specific CTLs have been associated with lower levels of blood urea nitrogen and creatinine, suggesting that HTNV-specific CTLs may help reduce the risk of progression to acute renal failure caused by HTNV infection and contribute to the reduction of disease severity (71, 72). Considering the prominent protective effect of epitope LL9 in kidneys of HLA-A2.1/K^b^ Tg mice, which is possibly attributable to the tendentious distributions of epitope-specific CTLs (73), LL9-based vaccination may induce effective CTL responses for the prevention of kidney damage caused by HTNV infection in humans.

Elucidating the cellular immune responses against immunodominant epitopes on HTNV is beneficial for the development and evaluation of candidate peptide vaccines. There are heterogeneous populations of CD8^+^ T cells after distinct virus-specific peptide stimulation characterized by different multi-cytokine secretion profiles and proliferation abilities (57, 74, 75). However, due to the limited number of cells, we did not perform more functional assays except the detection of IFN-γ secretion *in vitro* to confirm that these epitopes could induce functional CTLs. Since we identified and demonstrated that HTNV Gn-derived HLA-A*0201-restricted nonapeptide LL9 could act as immunodominant epitopes to induce effective immune responses against HTNV, we will further explore the characteristics of CTL responses elicited by epitope LL9 in our future study together with its structural biology by performing, for example, X-ray crystal structure analysis of the peptide LL9/HLA-A*0201 complex.

In summary, we are the first group to identify seven HTNV GP-derived HLA-A*0201-restricted nine-mer CTL epitopes with immunoreactivity, which may elicit protective IFN-γ-secreting CTL responses in patients with HFRS. Three chosen epitopes all exhibited immunogenicity characterized by significant inhibition of HTNV replication in HLA-A2.1/K^b^ Tg mice that received the peptide vaccination. More importantly, epitope LL9 showed a powerful ability to induce effective CTL responses against HTNV infection in major target organs of HLA-A2.1/K^b^ Tg mice, especially a prominent protective effect in kidneys, suggesting that LL9 was an immunodominant epitope that could elicit specific CLT responses to eliminate HTNV *in vivo*. These results provide novel valuable information to better understand CTL responses against HTNV infection, which may support the diagnosis and immune-targeting of HFRS, and promote the development of an effective peptide vaccine to prevent HTNV infection in humans.

## Ethics Statement

The study was approved by the Institutional Review Board of the Fourth Military Medical University, and all enrolled patients or their guardians signed an informed consent form before their blood was collected. The animal test was carried out in accordance with the recommendations of the Guide for the Care and Use of Laboratory Animals, the National Health and Medical Research Council of China. The protocol was approved by the Committee on the Ethics of Animal Experiments of the Fourth Military Medical University under license number XJYYLL-2014437.

## Author Contributions

The study was conceived and experiments designed by YM, FZ, and KT. The experiments were performed by KT, LC, and YM. Data were analyzed and interpreted by KT, LC, YM, BJ, and FZ. CZ, YusiZ, XZ, YunZ, and RZ contributed to the reagents, materials, or analytic tools. While KT and LC drafted the paper, all other authors critically revised the manuscript for important intellectual content.

## Conflict of Interest Statement

The authors declare that the research was conducted in the absence of any commercial or financial relationships that could be construed as a potential conflict of interest.

## Footnotes

^1^https://www-bimas.cit.nih.gov/molbio/hla_bind/.

^2^http://www.syfpeithi.de/0-Home.htm.

## Abbreviations

VFV9, peptide VLIEGKCFV; VV9, peptide VMASLVWPV; VPV9, peptide VLLSILCPV; SV9, peptide SLFSLLPGV; SL9, peptide SLTECPTFL; FL9, peptide FLLVLESIL; LL9, peptide LIWTGMIDL; VI9, peptide VIGQCIYTI.
