# Supplementary material for: Novel Identified HLA-A*0201-Restricted Hantaan Virus Glycoprotein Cytotoxic T-Cell Epitopes Could Effectively Induce Protective Responses in HLA-A2.1/Kb Transgenic Mice May Associate with the Severity of Hemorrhagic Fever with Renal Syndrome
Source: Front Immunol. 2017 Dec 12;8:1797. doi: 10.3389/fimmu.2017.01797 (PMC5732971; doi:10.3389/fimmu.2017.01797)
Supplement: Supplementary file 1 [file Data_Sheet_1.docx]

# Supplementary Table

## SUPPELEMENTARY TABLE 1: The median numbers of epitope-specific IFN-γ-secreting CTLs in different disease severity group.

|  | **VFV9** | | **VV9** | | **SV9** | | **SL9** | | **FL9** | | **LL9** | | **VI9** | |
| --- | --- | --- | --- | --- | --- | --- | --- | --- | --- | --- | --- | --- | --- | --- |
|  | **M/M** | **S/C** | **M/M** | **S/C** | **M/M** | **S/C** | **M/M** | **S/C** | **M/M** | **S/C** | **M/M** | **S/C** | **M/M** | **S/C** |
| **Median** | 101 | 47 | 55 | 23 | 133 | 36 | 61 | 57 | 60 | 33 | 62 | 62 | 34 | 38 |
| **IQR** | 75-127 | 24-70 | 25-150 | 23-134 | 70-195 | 28-43 | 35-90 | 30-206 | 3-117 | 13-52 | 50-87 | 45-106 | 7-60 | 0-76 |
| IQR, interquartile ranges; M/M, Mild/moderate; S/C, severe/critical. | | | | | | | | | | | | | | |

## SUPPELEMENTARY TABLE 2: Sensitivity and specificity of tetramer compared to HTNV serology testing.

| **Serology** | **Positive** | **Negative** |
| --- | --- | --- |
| **A. VFV9/HLA-A*0201 tetramer** | | |
| Positive | 15 | 1 |
| Negative | 11 | 9 |
| Cut-off value = 0.11%  Sensitivity = 57.7% Specificity = 90.0%  The exact 95% confidence interval estimates for sensitivity and specificity are (36.9%, 76.7%) and (55.5%, 99.8%), respectively. | | |
| **B. VV9/HLA-A*0201 tetramer** | | |
| Positive | 22 | 4 |
| Negative | 2 | 6 |
| Cut-off value = 0.04%  Sensitivity = 91.7% Specificity = 60.0%  The exact 95% confidence interval estimates for sensitivity and specificity are (73.0%, 99.0%) and (26.2%, 87.8%), respectively. | | |
| **C. SV9/HLA-A*0201 tetramer** | | |
| Positive | 16 | 1 |
| Negative | 11 | 9 |
| Cut-off value = 0.14%  Sensitivity = 59.3% Specificity = 90.0%  The exact 95% confidence interval estimates for sensitivity and specificity are (38.8%, 77.6%) and (55.5%, 99.8%), respectively. | | |
| **D. SL9/HLA-A*0201 tetramer** | | |
| Positive | 18 | 1 |
| Negative | 7 | 9 |
| Cut-off value = 0.19%  Sensitivity = 72.0% Specificity = 90.0%  The exact 95% confidence interval estimates for sensitivity and specificity are (50.6%, 87.9%) and (55.5%, 99.8%), respectively. | | |
| **E. FL9/HLA-A*0201 tetramer** | | |
| Positive | 18 | 1 |
| Negative | 7 | 9 |
| Cut-off value = 0.10%  Sensitivity = 72.0% Specificity = 90.0%  The exact 95% confidence interval estimates for sensitivity and specificity are (50.6%, 87.9%) and (55.5%, 99.8%), respectively. | | |
| **F. LL9/HLA-A*0201 tetramer** | | |
| Positive | 25 | 1 |
| Negative | 0 | 9 |
| Cut-off value = 0.09%  Sensitivity = 100% Specificity = 90.0%  The exact 95% confidence interval estimates for sensitivity and specificity are (86.3%, 100%) and (55.5%, 99.6%), respectively. | | |
| **G. VI9/HLA-A*0201 tetramer** | | |
| Positive | 24 | 1 |
| Negative | 1 | 9 |
| Cut-off value = 0.11%  Sensitivity = 96.0% Specificity = 90.0%  The exact 95% confidence interval estimates for sensitivity and specificity are (79.7%, 99.9%) and (55.5%, 99.8%), respectively. | | |
| Based on receiver operating characteristic (ROC) curve analysis, the values of optimal operating points (OOPs), which showed the largest positive likelihood ratio, were set as cut-off value. The frequency exceeded the cut-off value was defined as positive. | | |
